# BCG vaccination of healthcare workers for protection against COVID-19: 12-month outcomes from an international randomised controlled trial

**DOI:** 10.1016/j.jinf.2024.106245

**Published:** 2024-10

**Authors:** Nicole L. Messina, Laure F. Pittet, Ellie McDonald, Cecilia Moore, Simone Barry, Marc Bonten, Anthony Byrne, John Campbell, Julio Croda, Mariana G. Croda, Margareth Dalcolmo, Fernando F. de Almeida e Val, Roberto D. de Oliveira, Glauce dos Santos, Mark W. Douglas, Kaya Gardiner, Amanda Gwee, Bruno A. Jardim, Tobias Kollmann, Marcus VG Lacerda, Michaela Lucas, David J. Lynn, Laurens Manning, Helen Marshall, Abby O’Connell, Kirsten P. Perrett, Jeffrey J. Post, Cristina Prat-Aymerich, Jorge L. Rocha, Jesus Rodriguez-Baño, Ushma Wadia, Adilia Warris, Andrew Davidson, Nigel Curtis, the BRACE Trial Consortium Group

**Affiliations:** a Infectious Diseases Group, Murdoch Children's Research Institute, Parkville, Victoria, Australia; b Department of Paediatrics, The University of Melbourne, Parkville, Victoria, Australia; c Immunology, Vaccinology, Rheumatology and Infectious Diseases Unit, Geneva and University Hospitals and Faculty of Medicine, Geneva, Switzerland; d Clinical Epidemiology and Biostatistics Unit, Murdoch Children's Research Institute, Parkville, Victoria, Australia; e Precision Medicine Theme, South Australian Health and Medical Research Institute, Adelaide, South Australia, Australia; f Department of Thoracic Medicine, Royal Adelaide Hospital, Adelaide, South Australia, Australia; g Julius Center for Health Sciences and Primary Care, University Medical Centre Utrecht, Utrecht University, the Netherlands; h European Clinical Research Alliance on Infectious Diseases, Utrecht, Netherlands; i St Vincent's Hospitals, Darlinghurst, New South Wales, Australia; j Partners In Health, Socios En Salud, Peru; k Thoracic Society of Australia & New Zealand (NSW/ACT Branch), Australia; l Exeter Collaboration for Academic Primary Care, University of Exeter Medical School, Exeter, United Kingdom; m Fiocruz Mato Grosso do Sul, Fundação Oswaldo Cruz, Campo Grande, Mato Grosso do Sul, Brazil; n Department of Epidemiology of Microbial Diseases, Yale School of Public Health, New Haven, CT, USA; o Universidade Federal de Mato Grosso do Sul, Campo Grande, Mato Grosso do Sul, Brazil; p Centro de Referência Professor Hélio Fraga, ENSP/FIOCRUZ (Fundação Oswaldo Cruz), Rio de Janeiro, Brazil; q Tropical Medicine Foundation Dr Heitor Vieira Dourado, Manaus, Brazil; r State University of Mato Grosso do Sul, Dourados, Brazil; s Post Graduate Program in Health Sciences, Federal University of Grande Dourados, Dourados, Brazil; t Storr Liver Centre, The Westmead Institute for Medical Research, The University of Syndey at Westmead Hospital, Westmead, New South Wales, Australia; u Centre for Infectious Diseases and Microbiology, Sydney Infectious Diseases Institute, The University of Sydney at Westmead Hospital, Westmead, New South Wales, Australia; v Research Operations, The Royal Children's Hospital Melbourne, Parkville, Victoria, Australia; w Infectious Diseases, Royal Children’s Hospital Melbourne, Parkville, Victoria, Australia; x Antimicrobials Group, Murdoch Children’s Research Institute, Parkville, Victoria, Australia; y Institute of Clinical Research Carlos Borborema, Doctor Heitor Vieira Dourado Tropical Medicine Foundation, Manaus, Brazil; z Wesfarmers Centre for Vaccines and Infectious Diseases, Telethon Kids Institute, Nedlands, Western Australia, Australia; aa Instituto Leônidas & Maria Deane, Oswaldo Cruz Foundation Ministry of Health, Manaus, Brazil; ab University of Texas Medical Branch, Galveston, TX, USA; ac Department of Immunology, Pathwest, Queen Elizabeth II Medical Centre, Nedlands, Western Australia, Australia; ad Department of Immunology, Sir Charles Gairdner Hospital, Nedlands, Western Australia, Australia; ae Department of Immunology, Perth Children's Hospital, Nedlands, Western Australia, Australia; af School of Medicine, University of Western Australia, Perth, Western Australia, Australia; ag Flinders Health and Medical Research Institute, Flinders University, Bedford Park, South Australia, Australia; ah Department of Infectious Diseases, Fiona Stanley Hospital, Murdoch, Western Australia, Australia; ai The University of Adelaide and the Women’s and Children’s Health Network, Adelaide, SA, Australia; aj Exeter Clinical Trials Unit, University of Exeter, Exeter, United Kingdom; ak Population Allergy Group, Murdoch Children’s Research Institute, Parkville, Victoria, Australia; al Department of Allergy and Immunology, Royal Children's Hospital Melbourne, Parkville, Victoria, Australia; am Department of Infectious Diseases, Prince of Wales Hospital, Randwick, New South Wales, Australia; an School of Clinical Medicine, University of New South Wales, Sydney, New South Wales, Australia; ao Helio Fraga Reference Center, Oswaldo Cruz Foundation Ministry of Health, Curicica, Brazil; ap Division of Infectious Diseases and Microbiology, Department of Medicine, Hospital Universitario Virgen Macarena, University of Seville, Biomedicines Institute of Seville-Consejo Superior de Investigaciones Científicas, Seville, Spain; aq CIBER de Enfermedades Infecciosas, Instituto de Salud Carloss III, Madrid, Spain; ar Medical Research Council Centre for Medical Mycology, University of Exeter, Exeter, United Kingdom; as Department of Infectious Diseases, Great Ormond Street Hospital, London, United Kingdom; at Melbourne Children's Trial Centre, Murdoch Children’s Research Institute, Parkville, Victoria, Australia

**Keywords:** Bacille Calmette-Guérin (BCG) Vaccine, Randomised controlled trial, Breakthrough infection, COVID-19, Immunity

## Abstract

**Objectives:**

Bacille Calmette-Guérin (BCG) vaccine has immunomodulatory effects that may provide protection against unrelated infectious diseases. We aimed to determine whether BCG vaccination protects adults against COVID-19.

**Design:**

Phase III double-blind randomised controlled trial.

**Setting:**

Healthcare centres in Australia, Brazil, the Netherlands, Spain, and the United Kingdom during the COVID-19 pandemic.

**Participants:**

3988 healthcare workers with no prior COVID-19 and no contraindication to BCG.

**Intervention:**

Randomised 1:1 using a web-based procedure to receive a single 0.1 mL intradermal dose of BCG-Denmark (BCG group, n = 1999) or saline (placebo group, n = 1989).

**Main outcome measures:**

Difference in incidence of (i) symptomatic and (ii) severe COVID-19 during the 12 months following randomisation in the modified intention to treat (mITT) population (confirmed SARS-CoV-2 naïve at inclusion).

**Results:**

Of the 3988 participants randomised, 3386 had a negative baseline SARS-CoV-2 test and were included in the mITT population. The 12-month adjusted estimated risk of symptomatic COVID-19 was higher in the BCG group (22.6%; 95% confidence interval [CI] 20.6 to 24.5%) compared with the placebo group (19.6%; 95% CI 17.6 to 21.5%); adjusted difference +3.0% points (95% CI 0.2 to 5.8%; p = 0.04). The 12-month adjusted estimated risk of severe COVID-19 (mainly comprising those reporting being unable to work for ≥3 consecutive days) was 11.0% in the BCG group (95% CI 9.5 to 12.4%) compared with 9.6% in the placebo group (95% CI 8.3 to 11.1%); adjusted difference +1.3% points (95% CI −0.7 to 3.3%, p = 0.2). Breakthrough COVID-19 (post COVID-19 vaccination) and asymptomatic SARS-CoV-2 infections were similar in the two groups. There were 18 hospitalisations due to COVID-19 (11 in BCG group, 7 in placebo group; adjusted hazard ratio 1.56, 95% CI 0.60 to 4.02, p = 0.4) and two deaths due to COVID-19, both in the placebo group.

**Conclusions:**

Compared to placebo, vaccination with BCG-Denmark increased the risk of symptomatic COVID-19 over 12 months among healthcare workers and did not decrease the risk of severe COVID-19 or post-vaccination breakthrough COVID-19.

**Trial registration:**

ClinicalTrials.gov NCT04327206.

## Introduction

The bacille Calmette-Guérin (BCG) vaccine has greater protective effects than can be attributed to protection against tuberculosis alone. These beneficial off-target effects include reduction in all-cause mortality and protection against infectious diseases in infants in high-mortality settings,^1^, ^2^, ^3^, ^4^, ^5^ and reduced respiratory infectious disease in adults and the elderly.^6^, ^7^, ^8^ The off-target effects of BCG vaccination are proposed to result from the immunomodulatory effects of BCG, including the enhancement of immune responses to unrelated pathogens by the induction of trained innate immunity. 9, 10, 11

The emergence of the SARS-CoV-2 virus as a novel human pathogen resulted in the devastating COVID-19 pandemic. Given its documented protection against unrelated infectious diseases, BCG vaccination presented a potential early preventive measure to reduce the impact of COVID-19 until specific vaccines could be developed.^12^, ^13^

The *BCG vaccination to reduce the impact of COVID-19 in healthcare workers* (BRACE) trial aimed to determine if BCG-Denmark vaccination reduces the incidence and severity of COVID-19 in adult healthcare workers compared with placebo.^14^ We previously reported the primary outcomes of the BRACE trial, which showed that, compared with placebo vaccination, BCG vaccination did not reduce the incidence of COVID-19 in the six months following randomisation.^15^ Here we report the secondary outcomes of protection against symptomatic COVID-19, severe COVID-19, breakthrough COVID-19 (after COVID-19 vaccinations) and asymptomatic SARS-CoV-2 infection in the 12 months following randomisation.

## Methods

### Trial design and setting

The BRACE trial (ClinicalTrials.gov NCT04327206) is a multicentre phase III randomised controlled trial in healthcare workers. Participants were recruited from 36 sites in two stages. Stage 1 (recruitment March 2020 to May 2020) required influenza vaccination within 24 h of randomisation and recruited participants in Australia. Stage 2 (recruitment from May 2020 to April 2021) was double-blinded, placebo-controlled and recruited participants in Australia, the Netherlands, Spain, the United Kingdom and Brazil.

As pre-specified in the statistical analysis plan,^16^ due to negligible SARS-CoV-2 community transmission in Australia during follow-up for participants in Stage 1, COVID-19 primary and secondary outcomes in the BRACE trial were only analysed for participants in Stage 2 of the trial. The BRACE trial protocol and primary outcomes at six months have been previously published^14^, ^15^ and the statistical analysis plan^16^, ^17^ is available at mcri.figshare.

### Ethical approval and regulation

The BRACE trial was approved by the Royal Children’s Hospital Melbourne Human Research Ethics Committee (No. 62586); the protocol was approved by the ethics committee at each site and each participant provided informed consent. The trial was overseen by an independent data safety and monitoring board and a steering committee.

### Participants and eligibility criteria

Healthcare workers from participating centres were provided access to the participant information and consent form and invited to attend a baseline visit at which eligibility was ascertained. Exclusion criteria included: a previous positive test for SARS-CoV-2; contraindications to BCG vaccination; vaccination with BCG vaccine within the last year, any live-attenuated vaccine within the last month or any COVID-specific vaccine; and involvement in another COVID-19 prevention trial.

### Randomisation and blinding

Participants were randomised to the BCG or placebo group in a 1:1 ratio using a web-based procedure (REDCap®),^18^ in randomly permuted blocks of variable length. Randomisation was stratified by study stage, site, participant age and presence of comorbidity. Investigators, statisticians, and trial staff (except those involved in randomisation, vaccination, and safety monitoring) were blinded to the randomisation group throughout the trial.

### Intervention

Following randomisation, a single 0.1 mL dose of BCG-Denmark (AJ Vaccines, Copenhagen; corresponding to 2–8×10^5^ colony forming units of *Mycobacterium bovis*, Danish strain 1331) or saline placebo was administered by intradermal injection in the region of the deltoid muscle.

### Data collection

The REDCap platform was used for data collection.^18^ Participants were asked weekly if they had been unwell using a custom-built smartphone application (Trial Symptom Tracker, WeGuide)^19^ and/or by direct contact (phone call, text message). During each episode of illness, symptom reports were collected daily, and participants were requested to undergo SARS-CoV-2 respiratory swab testing. Detailed questionnaires were also completed at baseline and 3-monthly during follow-up. Reporting of COVID-19 vaccinations was done via the Trial Symptom Tracker application, during weekly direct contact follow-up and with 3-monthly surveys. Additional information on hospitalisations was obtained from medical records. Blood was collected at baseline, and 3, 6, 9, and 12 months after randomisation for measurement of anti-SARS-CoV-2 nucleocapsid antibodies (Roche Cobas Elecsys anti-SARS-CoV-2 assay)^20^ to determine SARS-CoV-2 exposure prior to randomisation (baseline seropositivity) and during the trial (seroconversion).

### Outcomes

Symptomatic COVID-19 was defined as an episode of illness with fever or at least one symptom of respiratory disease (sore throat, cough, or shortness of breath), and a positive SARS-CoV-2 test (PCR, rapid antigen test (RAT) or seroconversion). Severe COVID-19 was defined as an episode of illness with a positive SARS-CoV-2 test which resulted in at least one of the following for the participant: (i) death; (ii) hospitalisation; or (iii) being confined to bed (i.e. non-ambulant) or too unwell to go to work for three or more consecutive days.

Secondary outcomes included: any COVID-19 (symptomatic or severe); number of COVID-19 episodes; number of days with symptoms, absent from work or confined to bed due to COVID-19; occurrences of pneumonia, need for oxygen therapy, hospitalisation, admission to critical care, mechanical ventilation or death due to COVID-19; and asymptomatic SARS-CoV-2 infection; all assessed over the 12 months following randomisation.

Analyses of breakthrough COVID-19 infections were pre-specified prior to unblinding for symptomatic COVID-19 and severe COVID-19 (as defined above) occurring ≥14 days after the (i) first dose or (ii) primary course of any COVID-19 vaccine (Supplementary methods).

### Statistical analysis

The statistical analysis plan and addendum (which provides clarifying information for derivation of outcomes based on seroconversion) were finalised and made publicly available prior to analysis.^16^, ^17^ Analyses were done using Stata v16.1 (StataCorp, College Station, TX). The primary analyses estimating symptomatic and severe COVID-19 were done using a modified intention-to-treat (mITT) population, restricted to participants with a negative baseline SARS-CoV-2 test result. Incidence of symptomatic or severe COVID-19 were compared between the BCG and placebo groups using difference in proportions, estimated using a time-to-event analysis adjusted for the stratification factors used for randomisation. Participants were censored at 12 months or at the first occurrence of not being able to ascertain whether a COVID-19 episode had occurred (i.e. missing data for ≥3 consecutive days or episode of illness without a SARS-CoV-2 test result).

Pre-specified supplementary and sensitivity analyses were done to provide additional insights: (i) using the ITT population; (ii) using PCR/RAT results only (without serology) for defining COVID-19 episodes; and (iii) in the subset of the mITT population with scar data available, comparing participants in the BCG group who developed a BCG scar by 12 months to participants in the placebo group or in BCG group who did not develop a BCG scar.

Pre-specified sub-group analyses were done by: (i) presence of comorbidities (yes/no, for any comorbidity and by each comorbidity); (ii) age group (<40 years/40 to 59 years/≥60 years); (iii) geographical location (Brazil/Europe and Australia); (iv) sex (male/female); (v) history of previous BCG vaccination (BCG-naïve/previous BCG); (vi) presence of BCG scar at randomisation (BCG scar/no BCG scar) and (v) baseline serology results to SARS-CoV-2 (negative/non-negative).

The other outcome analyses were done using the mITT population and were adjusted for the stratification factors used for randomisation. Hazard ratios (HR) were used to compare the other time-to-event outcomes: any COVID-19 (symptomatic or severe), pneumonia, need for oxygen therapy, hospitalisation, admission to critical care, mechanical ventilation or death due to COVID-19. Incidence rate ratios (IRR) were used to compare quantitative outcomes: the number of episodes, days with symptoms, days unable to work, days confined to bed. Occurrence of asymptomatic SARS-CoV-2 infection was compared between the randomisation groups using difference in proportion estimated using a binomial regression model, adjusted for stratification factors used at randomisation. The analysis population for the latter was restricted to participants in the mITT who did not receive CoronaVac (as this vaccine induces SARS-CoV-2 nucleocapsid antibodies) and with data available (i.e. serology data not unavailable/indeterminant and no missing COVID-19 episode data).

The analysis on occurrences of breakthrough infections was analysed in the ITT population but restricted to participants who received COVID-19 vaccinations during the trial. Hazard ratios (HR) were estimated using a Cox regression analysis model adjusted for (i) stratification factors used during randomisation, (ii) type of vaccine received and (iii) COVID-19 infection (symptomatic or severe) prior to COVID-19 vaccine receipt.

## Results

**Fig. 1:**
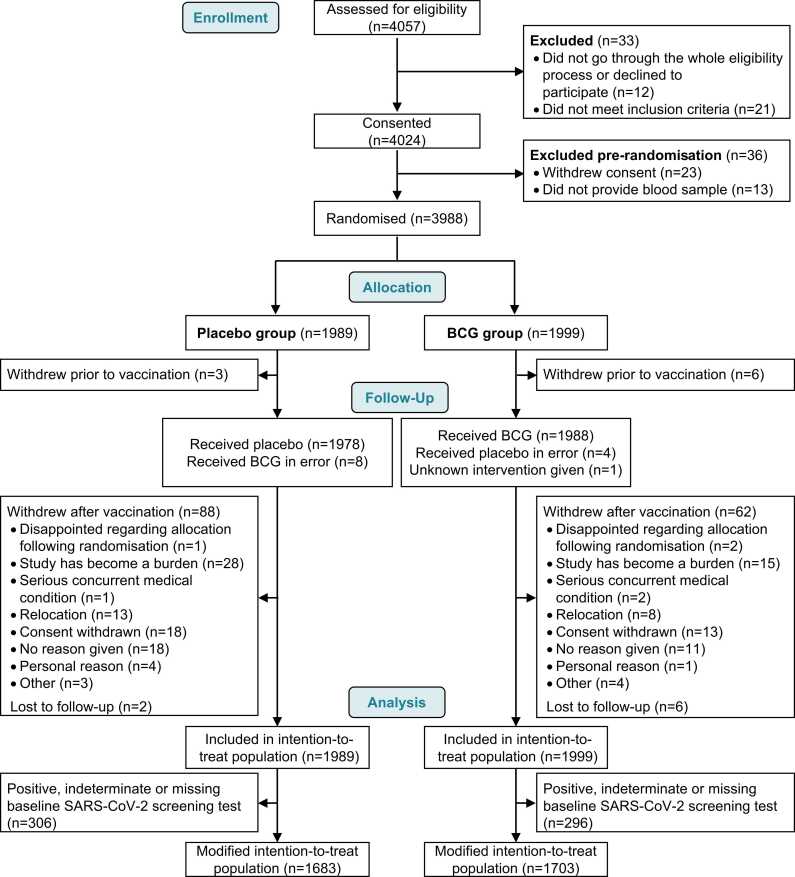
Participant CONSORT diagram. Baseline SARS-CoV-2 screening result based on serology or respiratory swab PCR testing (Brazil only) at baseline. PCR, polymerase chain reaction.

A total of 3988 healthcare workers were included in the BRACE trial between May 14th 2020 and April 1st 2021 and randomised to receive BCG (1999 participants) or placebo (1989 participants) vaccination (Fig. 1). Baseline characteristics were similar between the two groups (Table 1 and Supplementary Table 1) except for a marginally lower proportion of females in the BCG group. At baseline, SARS-CoV-2 serology was positive in 14.1% of all participants and SARS-CoV-2 respiratory swabs were positive in 2.7% of participants from Brazil (and inconclusive or missing in 0.5%). Overall, the mITT population included 84.9% of randomised participants (1683 in placebo group and 1703 in BCG group) with negative baseline SARS-CoV-2 test results. Overall, 96.3% of participants were followed up for 12 months; withdrawals were slightly higher in the placebo group (4.6% vs 3.4%). COVID-19 vaccinations were similar between the two groups with an overall median time to first COVID-19 vaccination of 2.7 months (interquartile range [IQR] 1.5, 5.6) from randomisation (Table 1).

**Table 1:** Participant baseline characteristics and COVID-19 vaccinations.

	Intention-to-treat population		Modified intention-to-treat population	
	Placebo	BCG	Placebo	BCG
Baseline characteristic	N = 1989	N = 1999	N = 1683	N = 1703
Sex				
Male	495 (25%)	550 (28%)	402 (24%)	455 (27%)
Female	1494 (75%)	1446 (72%)	1281 (76%)	1245 (73%)
Declined	0 (0%)	3 (0%)	0 (0%)	3 (0%)
Age				
<40 years old	914 (46%)	916 (46%)	734 (44%)	740 (43%)
40−59 years old	922 (46%)	925 (46%)	803 (48%)	812 (48%)
≥60 years old	153 (8%)	158 (8%)	146 (9%)	151 (9%)
Any comorbidity^a^	675 (35%)	702 (36%)	560 (34%)	596 (36%)
Diabetes	74 (4%)	62 (3%)	67 (4%)	52 (3%)
Type 1	8 (11%)	10 (16%)	8 (12%)	7 (13%)
Type 2	56 (76%)	49 (79%)	52 (78%)	43 (83%)
Other/unsure	10 (14%)	3 (5%)	7 (10%)	2 (4%)
Cardiovascular disease	250 (13%)	263 (13%)	214 (13%)	235 (14%)
Hypertension	236 (12%)	249 (12%)	200 (12%)	222 (13%)
Ischaemic heart disease	7 (0%)	5 (0%)	7 (0%)	5 (0%)
Congestive heart disease	2 (0%)	0 (0%)	2 (0%)	0 (0%)
Other cardiovascular disease	13 (1%)	19 (1%)	12 (1%)	18 (1%)
Chronic respiratory disease	108 (5%)	126 (6%)	92 (5%)	111 (7%)
Obesity^b^	417 (21%)	441 (22%)	338 (21%)	361 (22%)
Missing	48	33	45	32
BCG vaccination in the past	1526 (77%)	1543 (77%)	1248 (74%)	1267 (74%)
BCG scar at randomisation	1355 (76%)	1356 (76%)	1105 (73%)	1113 (73%)
Missing	201	213	167	180
Geographical region				
Australia	206 (10%)	216 (11%)	206 (12%)	214 (13%)
Europe	500 (25%)	498 (25%)	478 (28%)	483 (28%)
South America	1283 (65%)	1285 (64%)	999 (59%)	1006 (59%)
**COVID-19 vaccination**				
Any COVID-19 vaccination during the trial	1920 (97%)	1935 (97%)	1630 (97%)	1648 (97%)
1 vaccination	153 (8%)	152 (8%)	134 (8%)	137 (8%)
2 vaccinations	974 (49%)	979 (49%)	841 (50%)	848 (50%)
3 vaccinations	793 (40%)	804 (40%)	655 (39%)	663 (39%)
Months between randomisation and first COVID-19 vaccination, median [IQR]	2.6 [1.5, 5.5]	2.7 [1.5, 5.6]	2.7 [1.6, 5.9]	2.8 [1.6, 6.0]
Type of first COVID-19 vaccination received				
AstraZeneca/Oxford (ChAdOx1-S, Covishield)	623 (32%)	659 (34%)	540 (33%)	563 (34%)
Pfizer/BioNTech (BNT162b2, Comirnaty)	433 (23%)	444 (23%)	391 (24%)	406 (25%)
Moderna (mRNA-1273)	117 (6%)	108 (6%)	110 (7%)	107 (6%)
Sinovac (CoronaVac)	676 (35%)	651 (34%)	523 (32%)	502 (30%)
Johnson & Johnson (Ad26. COV2. S)	71 (4%)	73 (4%)	66 (4%)	70 (4%)
Missing	69	64	53	55
Type of primary series of COVID-19 vaccination(s) received				
Autologous	1827 (92%)	1845 (92%)	1552 (92%)	1572 (92%)
Heterologous	11 (0%)	9 (0%)	10 (1%)	7 (0%)
Missing	151	145	121	124

IQR, interquartile range.

a Diabetes, cardiovascular disease, chronic respiratory disease or obesity^b^.

b Body mass index (weight in kilograms divided by the square of the height in metres) ≥30 kg/m^2^.

**Fig. 2:**
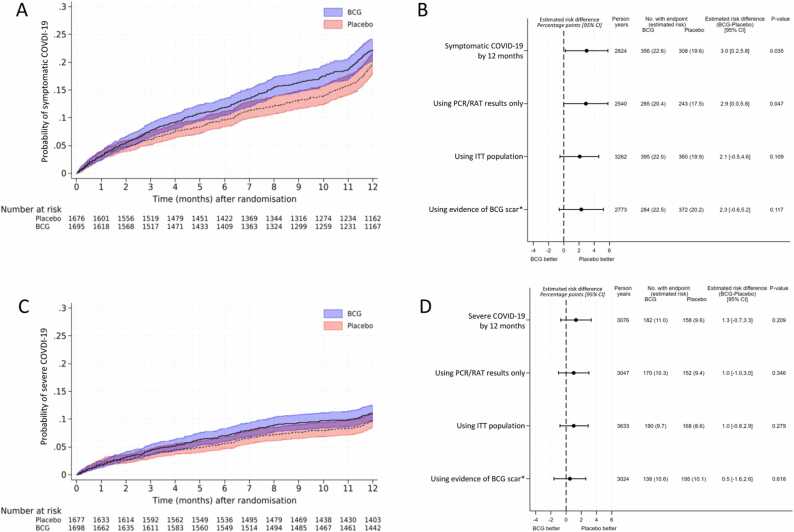
COVID-19 by 12 months following randomisation, by treatment arm. Panels A and B present symptomatic COVID‐19 and panels C and D present severe COVID‐19. Panels A and C present Kaplan–Meier curves with 95% confidence intervals in shaded areas (unadjusted analyses in the modified intention‐to‐treat population (mITT)). Panels B and D present forest plots of the between‐group difference in the percentage of participants with symptomatic or severe COVID‐19, with 95% confidence intervals (adjusted for stratification factors used in randomisation; primary, sensitivity, and supplementary analyses). *Population analysed was the subset of the mITT population with scar data available at 12 months, the treatment arms for this analysis are placebo or BCG without a scar evident at 12 months vs BCG with a scar evident at 12 months. COVID-19, coronavirus disease 2019; ITT, intention-to-treat; PCR, polymerase chain reaction; RAT rapid antigen test.

In the 12 months following randomisation, symptomatic COVID-19 occurred in 308 participants in the placebo group (adjusted estimated risk 19.6%; 95% confidence interval [CI] 17.6 to 21.5%) and 356 participants in the BCG group (adjusted estimated risk 22.6%; 95% CI 20.6 to 24.5%), a 3.0% point (95% CI 0.2 to 5.8%, p = 0.04) difference, equating to a 15.3% increased relative risk of symptomatic COVID-19 in the BCG group compared to the control group (Table 2, Fig. 2). Severe COVID-19, as defined in the trial (death, hospitalisation, non-ambulant or too unwell to go to work for three or more consecutive days), occurred in 340 participants. The majority of severe COVID-19 cases were due to being non-ambulant (18%) or too unwell to go to work (77%) for three or more consecutive days. This represents a 1.3% point difference (95% CI −0.7 to 3.3%, p = 0.2) in severe COVID-19 between the placebo group (158 participants, adjusted estimated risk 9.6%; 95% CI 8.2 to 11.1%) and BCG group (182 participants, adjusted estimated risk 11.0%; 95% CI 9.5 to 12.4%) (Table 2, Fig. 2). The risk differences for symptomatic and severe COVID-19 were similar in supplementary and sensitivity analyses (Fig. 2) and with adjustment for sex (Supplementary Table 2).

**Table 2:** COVID-19 by 12 months following randomisation.

	Placebo	BCG	Difference^a^	P-value
	N = 1683	N = 1703	(BCG-Placebo)	
**Symptomatic COVID-19 by 12 months**	308	356		
Event rate per 100 person-years (95% CI)	21.7 (19.4 to 24.3)	25.2 (22.7 to 28.0)		
Adjusted estimated percent (95% CI)^a^	19.6% (17.6 to 21.5)	22.6% (20.6 to 24.5)	3.0% (0.2 to 5.8)	0.04
**Severe COVID-19 by 12 months**	158	182		
Hospitalised, n	7	11		
Non-ambulant for ≥3 consecutive days, n	34	26		
Too unwell to work for ≥3 consecutive days, n	117	145		
Event rate per 100 person-years (95% CI)	10.4 (8.9 to 12.1)	11.8 (10.2 to 14.0)		
Adjusted estimated percent (95% CI)^a^	9.6% (8.2 to 11.1)	11.0% (9.5 to 12.4)	1.3% (−0.7 to 3.3)	0.2
**Other outcomes by 12 months**				
Any COVID−19 (symptomatic or severe), n%	316 (18.8%)	366 (21.5%)	aHR 1.18 (1.02 to 1.38)	0.03
Pneumonia due to COVID−19, n%	9 (0.5%)	17 (1.0%)	aHR 1.88 (0.84 to 4.22)	0.1
Hospitalisation due to COVID−19, n%	7 (0.4%)	11 (0.6%)	aHR 1.56 (0.60 to 4.02)	0.4
Oxygen therapy due to COVID−19, n%	4 (0.2%)	11 (0.6%)	aHR 2.74 (0.87 to 8.61)	0.08
Admission to critical care due to COVID−19, n%	2 (0.1%)	5 (0.3%)	aHR 2.47 (0.48 to 12.72)	0.3
Mechanical ventilation due to COVID−19, n%	1 (0.1%)	2 (0.1%)	-	-
Death due to COVID−19, n%	2 (0.1%)	0	-	-
Number of days confined to bed due to COVID−19, median (IQR)^b^	4.0 (2.0 to, 7.0)	2.0 (1.0, 6.5)	aIRR 0.94 (0.67 to 1.31)	0.7
Number of episodes of COVID−19, median (IQR)^b^	1.0 (1.0 to 1.0)	1.0 (1.0 to 1.0)	aIRR 0.97 (0.83 to 1.12)	0.6
Asymptomatic SARS-CoV−2 infection, n/total n^c^	23/1060	32/1703		
Adjusted estimated percent (95% CI)^a^	2.45 (1.48 to 3.42)	3.12 (2.07 to 4.16)	0.67 (−0.76 to 2.10)	0.4
**Subgroup analysis of outcomes by 12 months with evidence of interaction with stratification factor**^d^				
**Number of days with symptoms,** median (IQR)^b^				
Age group randomisation strata				<0.001^e^
<40 years old	12.0 (8.0 to 19.0)	12.0 (8.0 to 19.0)	aIRR 0.88 (0.74 to 1.03)	
40 to 59 years old	13.0 (9.0 to 22.0)	13.0 (9.0 to 22.0)	aIRR 1.09 (0.89 to 1.34)	
≥60 years old	14.5 (8.0 to 32.0)	12.0 (8.0 to 18.0)	aIRR 0.44 (0.27 to 0.71)	
Comorbidity randomisation strata				0.003^e^
Absence of any comorbidity	12.0 (8.0 to 20.0)	12.0 (8.0 to 18.0)	aIRR 0.85 (0.74 to 0.97)	
Presence of any comorbidity	14.0 (9.0 to 22.0)	15.0 (8.0 to 23.0)	aIRR 1.26 (0.91 to 1.75)	
**Number of days unable to work,** median (IQR)^b^				
Age group randomisation strata				<0.001^e^
<40 years-old	7.0 (4.0 to 10.5)	5.0 (3.0 to 9.0)	aIRR 0.80 (0.65 to 0.98)	
40 to 59 years old	7.0 (3.0 to 12.0)	7.0 (3.0 to 10.0)	aIRR 1.17 (0.86 to 1.58)	
≥60 years old	10.0 (7.0 to 14.0)	4.0 (3.0 to 7.0)	aIRR 0.33 (0.19 to 0.59)	
Comorbidity randomisation strata				0.04
Absence of any comorbidity	7.0 (4.0 to 11.0)	6.0 (3.0 to 9.0)	aIRR 0.82 (0.68 to 1.00)	
Presence of any comorbidity	7.0 (4.0 to 11.0)	7.0 (3.0 to 12.0)	aIRR 1.13 (0.72 to 1.78)	
Breakthrough COVID-19 by 12 months				
Symptomatic COVID-19				
After 1st COVID vaccination**,** n/total n^f^	244 /1920	251 /1935		
Event rate per 100 person-years (95% CI)	21.0 (18.5 to 23.8)	21.8 (19.3 to 24.7)	aHR 1.05 (0.88 to 1.26)^g^	0.6
After primary series of COVID vaccination**,** n/total n^h^	208/1838	205/1855		
Event rate per 100 person-years (95% CI)	22.0 (19.2 to 25.2)	22.1 (19.2 to 25.3)	aHR 1.03 (0.85 to 1.25)^g^	0.8
Severe COVID-19				
After 1st COVID vaccination**,** n/total n^f^	102/1920	105/1935		
Hospitalised	2 (2%)	6 (6%)		
Non-ambulant	16 (16%)	14 (13%)		
Too unwell to work	84 (82%)	85 (81%)		
Event rate per 100 person-years (95% CI)	8.1 (6.7 to 9.8)	8.3 (6.8 to 10.0)	aHR 1.05 (0.8 to 1.38)^g^	0.7
After primary series of COVID vaccination**,** n/total n^h^	80/1838	77/1855		
Hospitalised	2 (2%)	3 (4%)		
Non-ambulant	9 (11%)	7 (9%)		
Too unwell to work	69 (86%)	67 (87%)		
Event rate per 100 person years (95% CI)	7.8 (6.3 to 9.8)	7.6 (6.1 to 9.5)	aHR 1.00 (0.73 to 1.37)^g^	1.0

aHR, adjusted Hazard Ratio (BCG/Placebo); aIRR, adjusted incidence rate ratio (BCG/Placebo); CI, confidence interval; IQR, interquartile range; n, number.

a Adjusted for stratification factors.

b Among participants with one or more episode of severe or symptomatic COVID-19.

c Complete case analysis: modified intention-to-treat (mITT) population with complete 0–12-month symptom data, without CoronaVac receipt and ≤9 months gap between blood samples.

d Due to a significant interaction between treatment arm and stratification factors, the number of days with symptoms, the number of days unable to work, and the number of unplanned absenteeism are only presented by subgroups.

e P-value for interaction between treatment arm and subgroup.

f Among participants in ITT with at least one COVID-19 vaccination reported by 12 months.

g Adjusted for stratification factors, type of vaccine received and prior symptomatic/severe COVID-19.

h Among participants in ITT with a primary series if COVID-19 vaccination (autologous or heterologous) reported by 12 months.

**Fig. 3:**
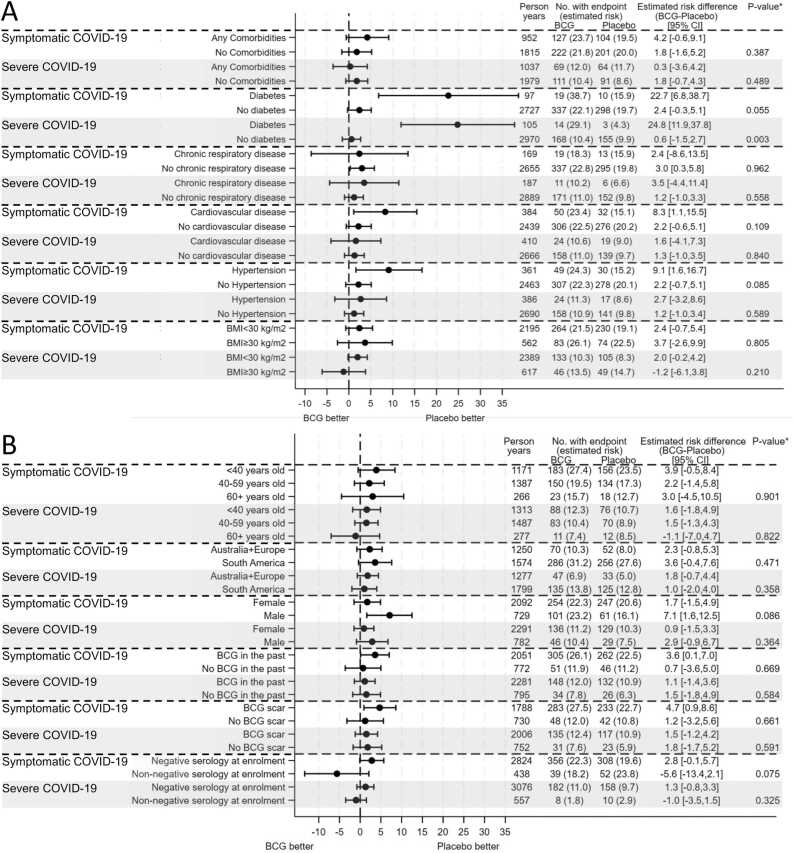
COVID-19 by 12 months following randomisation among subgroups. Forest plots of the between‐group difference in the percentage of participants with symptomatic or severe COVID‐19 with (A) comorbidity and (B) other baseline characteristic sub groups. Data are adjusted for stratification factors used in randomisation and are presented with 95% confidence intervals. *p-value presented is for the interaction between treatment arm and subgroup is presented. BMI, body mass index; COVID-19, coronavirus disease 2019.

In the pre-specified subgroup analysis, there was evidence of an interaction between treatment arm and presence of diabetes on the risk of severe and symptomatic COVID-19 (Fig. 3). Among the 119 participants with diabetes, there was a greater risk of severe COVID-19 for participants in the BCG group compared to the control group (adjusted estimated risk difference +24.8% points; 95% CI 11.9 to 37.8%), while no difference was seen in risk of severe COVID-19 among non-diabetic participants (adjusted estimated risk difference +0.6% point; 95% CI −1.5 to 2.7%). There was also a greater risk of symptomatic COVID-19 (+22.7% points; 95% CI 6.8 to 38.7%) in the BCG group compared to the control group for participants with diabetes (Fig. 3, Supplementary Table 3). There was no significant interaction observed between treatment arm and any other comorbidity or demographic subgroups on the risk of severe or symptomatic COVID-19 (Fig. 3).

The risk of any COVID-19 episode in the 12 months following randomisation was greater in the BCG group compared to the placebo group (adjusted hazard ratio [aHR] 1.18; 95% CI 1.02 to 1.38, p = 0.03). The risk of requiring oxygen therapy due to COVID-19 was greater in the BCG group compared to the placebo group although the confidence interval was wide and included no difference between the two groups (aHR 2.74; 95% CI 0.87 to 8.61; Table 2). There were two deaths due to COVID-19, both in the placebo group, and three participants who required mechanical ventilation due to COVID-19 (two in BCG group who survived, one in placebo group who died). There was no evidence of a difference between the BCG and placebo groups in the risk of other complications of COVID-19 (pneumonia, hospitalisation, admission to critical care), number of episodes of COVID-19, number of days confined to bed or the asymptomatic SARS-CoV-2 infections.

When comparing the number of days with symptoms and number of days unable to work between the BCG and placebo groups, there was evidence of an interaction between treatment arms and two randomisation strata (age group and comorbidities), which rendered an overall comparison between randomisation groups non-interpretable. In the ≥60-year age group, the participants in the BCG group had fewer days with symptoms compared with those in the placebo group (Table 2) (adjusted incidence rate ratio [aIRR] 0.44; 95% CI 0.27 to 0.71; interaction p < 0.001), while no difference between treatment groups was seen in the <40- and 40–59-year age groups. Among participants 60 years of age or older, the participants in the BCG group also had fewer days unable to work (aIRR 0.33; 95% CI 0.19 to 0.59; interaction p < 0.001) compared with those in the placebo group. Compared to the placebo group, the BCG group also had fewer days unable to work (aIRR 0.80; 95% CI 0.65 to 0.98) among participants younger than 40 years of age. Among participants without a comorbidity, the BCG group had fewer days with symptoms (aIRR 0.85; 95% CI 0.74 to 0.97, interaction p = 0.003) and unable to work (aIRR 0.82; 95% CI 0.68 to 1.00; interaction p = 0.04) than the placebo group. While among participants with a comorbidity, no difference was seen between arms in terms of number of days with symptoms or being unable to work.

Symptomatic COVID-19 occurred in 495 participants after the first dose of a COVID-19 vaccine and in 413 participants after completing the primary course of COVID-19 vaccinations. Severe COVID-19 occurred in 207 participants after the first dose of a COVID-19 vaccine and in 157 participants after the primary course of COVID-19 vaccinations. There was no evidence of a difference between the BCG and placebo groups in the risk of breakthrough symptomatic or severe COVID-19 after the first dose or primary course of COVID-19 vaccines (Table 2, Supplementary Figure 1).

Details on adverse events have been published previously.^15^, ^21^, ^22^ Overall, there were four participant deaths during the trial, all in the placebo group: two due to COVID-19 (reported above), one due to pneumonia unrelated to COVID-19 and one due to haemorrhagic-type stroke. There were no safety concerns following BCG vaccination in the BRACE trial.

## Discussion

In this international, double-blind, randomised controlled trial in healthcare workers, vaccination with BCG-Denmark increased the risk of symptomatic, but not severe, COVID-19 compared to placebo vaccination.

Due to the potential for BCG vaccination to provide protection against unrelated respiratory infectious disease, multiple trials have investigated whether BCG vaccination protects against COVID-19. Of the 13 trials published to date,^23^, ^24^ only the study nested in an ongoing trial investigating whether repeated doses of BCG-Japan vaccine improve glycaemic control in type 1 diabetic patients,^25^, ^26^ found a protective effect of BCG vaccination against COVID-19 (with 1/96 and 22/93 cases in the BCG group versus 6/48 and 20/48 cases in the control group in the first 15 months and proceeding 19 months respectively). In the 11 remaining trials, no statistically significant effect of BCG vaccination on COVID-19 was found. However, the three largest trials (each with over 2000 participants), including the BRACE trial 6-month primary outcome analysis, did suggest an increased risk of COVID-19 in BCG-vaccinated participants compared to placebo controls.^15^, ^27^, ^28^ However, none of these differences reached statistical significance.

Counterintuitively, in our study, the increased risk of COVID-19 in the BCG group was not accompanied by a corresponding decrease in asymptomatic infections in this group compared to the placebo group. Overall, there was a low asymptomatic infection rate observed in the BRACE trial (2.5% in the placebo group) compared to previous studies (between 12.6% and 39%).^29^, ^30^, ^31^, ^32^ This may have been due to weekly prospective follow-up which ensured that even short and mildly symptomatic episodes of illness were detected, while they may have been overlooked in studies with less frequent follow-up contact. In addition, use of positive serology to define COVID-19 episodes where PCR/RAT testing was negative or missing may have reduced our detection of asymptomatic episodes by misclassifying as COVID-19 a non-COVID-19 respiratory/febrile illnesses occurring in the same seroconversion period as an asymptomatic SARS-CoV-2 infection.

The clinical off-target effects of BCG vaccination are proposed to be underpinned by modulation of immune responses, including the induction of trained innate immunity. In adults, BCG vaccination results in increased in vitro cytokine responses to unrelated bacterial and fungal pathogens.^9^, ^33^ However, this effect is not consistently observed for viral pathogens, even in the presence of enhanced viral clearance.^33^, ^34^, ^35^ Few studies have investigated the effects of BCG vaccination on immune responses to SARS-CoV-2 in humans. An immunological sub-study involving BRACE trial participants from Australia showed that BCG vaccination reduced in vitro cytokine responses to SARS-CoV-2 and increased effector CD4^+^ and CD8^+^ T cells three months after randomisation.^36^ Although it was initially hypothesised that this effect of BCG might reduce pathogenic cytokine storm in severe COVID-19, it is also possible that the reduced cytokine responses prevent early SARS-CoV-2 clearance by the innate immune system thus resulting in increased symptomatic disease.^37^ Whether there may be a beneficial effect in protecting against hospitalisation or death from COVID-19 remains to be determined as the BRACE trial and other RCTs of BCG have been inadequately powered to assess this outcome.^23^

The effect of BCG vaccination on the subset of participants with diabetes in our trial contrasts with a recent trial of repeat BCG vaccine doses among diabetics. In that study, nested in an ongoing trial investigating whether multiple doses of BCG-Japan improve glycaemic control in type 1 diabetic patients, BCG-vaccinated participants had a reduced cumulative incidence of COVID-19 compared to placebo-vaccinated participants.^25^, ^26^ The contrasting effects of BCG vaccination on COVID-19 in diabetics observed between these two studies may be due to differences in BCG vaccine dosing or strain (1 dose of BCG-Denmark compared to 3–6 doses of BCG-Japan) and/or the difference in the commencement of follow-up period (immediate compared to 2.5–3 years after randomisation). In a small retrospective study of COVID-19 in patients in Qatar (n = 67), those with type 2 diabetes had more moderate-severe COVID-19 if they had a history of prior BCG vaccination (25/26, 96%) compared to those without prior BCG (5/8, 63%, p < 0.05), an effect that was not seen among non-diabetic patients.^38^ That study also found differences in serum metabolites between BCG-vaccinated and BCG-naïve diabetics, however, as samples were taken during the COVID-19 episode, whether these metabolic changes are a contributor or consequence of disease severity is unclear. In our trial, the diabetes subgroup included both type 1 and type 2 diabetics, however the effect of BCG did not appear to be limited to either type of diabetes.

BCG vaccination has been reported to alter immune responses to unrelated vaccines.^11^, ^39^ Therefore, as COVID-19 vaccinations became available during this trial we were able to also determine whether BCG vaccination altered the efficacy of COVID-19 vaccinations. Our finding that BCG vaccination did not influence protection against breakthrough COVID-19 is consistent with immunological sub-studies of RCTs which have shown no difference between BCG-vaccinated and placebo-vaccinated participants in anti-spike and/or anti-receptor binding domain (RBD) specific IgG responses to CoronaVac, ChAdOx1-S or BNT162b2 vaccination.^40^, ^41^ A small cohort study in India also found no effect of BCG re-vaccination on humoral responses to ChAdOx1-S, although some differences in T cell responses were observed.^42^

Strengths of our trial include the large size, recruitment in 36 sites across five countries, blinding of group allocation, stringent COVID-19 case definitions, close active follow-up of participants with daily data collection during illnesses and 3-monthly serology tests. Compared to the BRACE trial COVID-19 outcomes at 6 months post-randomisation, the increased follow-up time to 12 months increased the power of the trial and reduced variability in the observed effect of BCG vaccination.

The main limitation of our trial was the lower than planned participant recruitment due to the earlier-than-anticipated availability of COVID-19 vaccines. As healthcare workers, participants in the BRACE trial were among the first to receive COVID-19 vaccines as they became available in their respective regions. This had a large impact on the power for analysis of the more severe outcomes of hospitalisation or death due to COVID-19 in our trial due to the low incidence of these events, and the protection against hospitalisation or death afforded by COVID-19 vaccinations. Another limitation of the trial is the inclusion of ‘too sick to go to work’ or ‘unable to get out of bed’ for ≥3 consecutive days in the definition of severe COVID-19, representing 77% and 18% of participants with this outcome, respectively; only 5% of the participants with severe COVID-19 were hospitalised. Finally, although the study was placebo-controlled, robust blinding is a challenge in BCG trials due to the injection site reaction and scarring that develops in most people. This limitation was mitigated by use of objective outcomes (i.e. requiring a positive SARS-CoV-2 respiratory PCR/RAT test or seroconversion) and blinding of study staff involved with data collection and analysis.

In conclusion, vaccination with BCG-Denmark increased the risk of symptomatic, but not severe COVID-19 in healthcare workers. The effect on hospitalisation or death could not be determined. A differential effect of BCG vaccination on COVID-19 was found in participants with diabetes as well as on the duration of COVID-19 episodes in relation to age and presence of comorbidity. BCG vaccination did not reduce the risk of breakthrough COVID-19 after COVID-19 vaccinations.

## Funding

The trial is supported by the 10.13039/100000865Bill & Melinda Gates Foundation [INV-017302], the Minderoo Foundation [COV-001], Sarah and Lachlan Murdoch, the Royal Children’s Hospital Foundation [2020-1263 BRACE Trial], Health Services Union NSW, the 10.13039/501100014093Peter Sowerby Foundation, SA Health, the Insurance Advisernet Foundation, the NAB Foundation, the Calvert-Jones Foundation, the Modara Pines Charitable Foundation, the UHG Foundation Pty Ltd, Epworth Healthcare and individual donors. The funders had no role in the collection, analysis and interpretation of data or in the preparation, review or approval of the manuscript. The Murdoch Children’s Research Institute (MCRI) leads the BRACE trial across 36 sites in five countries. It is supported by the Victorian Government’s Operational Infrastructure Support Programme. NC, AG and KPP are supported by a 10.13039/501100000925National Health and Medical Research Council (NHMRC) Investigator Grants [GNT1197117, GNT1194694, GNT2008911]. DJL is supported by an EMBL Australia Group Leader award. HSM is supported by a NHMRC Practitioner Fellowship. LFP is supported by the 10.13039/501100001711Swiss National Science Foundation (Early Postdoc Mobility Grant, P2GEP3_178155). KPP is supported by a Melbourne Children’s Clinician-Scientist Fellowship. AW is supported by the UK Medical Research Council (MR/N006364/2).

## Author contributions

All authors contributed substantially to the BRACE trial. NC is the chief principal investigator of the BRACE trial. The statistical analysis plan was prepared with input from CM, NLM, NC, EMD, LFP and CM did all the statistical analysis. NLM wrote the first draft of the report, and all authors critically reviewed it. All authors had final responsibility for the decision to submit for publication. EMD and CM have directly accessed and verified the underlying data reported in the manuscript. NC attests that all listed authors meet authorship criteria and that no others meeting the criteria have been omitted.

## Declaration of Competing Interest

The authors declare the following financial interests/personal relationships which may be considered as potential competing interests: The trial is financially supported by the Foundations listed in the Funding section. Authors disclose funding support over the past 36 months: National Health and Medical Research Council (NHMRC) Ideas Grant (NM), NHMRC Investigator Grant (NC), Melbourne Children’s Clinician-Scientist Fellowship Grant (KPP). Outside of the submitted work, JCr has received grants or contracts from Sanofi, MSD & CEPI; payment or honoraria for presentations from Pfizer and participates on Latin American data safety monitoring/advisory boards for mRNA-1273 (Modern/Zodiac), RSV maternal vaccine (Pfizer), Qdenga vaccine (Takeda) and Nirmatrelvir/Ritonavir-Paxlovid (Pfizer). KG is a member of the Royal Children’s Hospital (RCH) Human Research Ethics Committee (the primary ethics committee providing approval for the BRACE trial) and Director of Research Operations at RCH; she abstained from all discussion, voting, approval and review related to the BRACE trial. All other authors declare no conflict of interest.

## Data Availability

Deidentified participant data and data dictionary are available to others on request and on completion of a signed data access agreement. Requests can be made in writing to braceresearch@mcri.edu.au.
