# Supplementary material for: BCG vaccination of healthcare workers for protection against COVID-19: 12-month outcomes from an international randomised controlled trial
Source: J Infect. 2024 Oct;89(4):None. doi: 10.1016/j.jinf.2024.106245 (PMC11409612; doi:10.1016/j.jinf.2024.106245)
Supplement: Supplementary file 1 — Supplementary material [file mmc1.docx]

**BCG vaccination of healthcare workers for protection against COVID-19: 12 month outcomes from an international randomised controlled trial**

**Supplementary Appendix**

[BRACE Trial Consortium Group 2](#_Toc163414396)

[Supplementary Methods 6](#_Toc163414397)

[Supplementary Table 1 8](#_Toc163414398)

[Supplementary Table 2 9](#_Toc163414399)

[Supplementary Table 3 9](#_Toc163414400)

[Supplementary Figure 1 10](#_Toc163414401)

BRACE Trial Consortium Group

**Australia (Victoria)**

MCRI Central Team: **Prof Nigel Curtis**, Prof Andrew Davidson, Kaya Gardiner, A/Prof Amanda Gwee, Tenaya Jamieson, Dr Nicole Messina, Thilanka Morawakage, Dr Susan Perlen, A/Prof Kirsten Perrett, Dr Laure Pittet, Amber Sastry, Jia Wei Teo;

Biostatisticians: **Francesca Orsini**, Prof Katherine Lee, Dr Cecilia Moore, Suzanna Vidmar;

Data Team: **Dr Laure Pittet**, Rashida Ali, Ross Dunn, Peta Edler, Grace Gell, Casey Goodall, Richard Hall, Ann Krastev, Dr Nathan La, Dr Ellie McDonald, Nick McPhate, Thao Nguyen, Jack Ren, Luke Stevens;

Laboratory Core Team: **Dr Nicole Messina**, Ahmed Alamrousi, Rhian Bonnici, Dr Thanh Dang, Susie Germano, Jenny Hua, Rebecca McElroy, Monica Razmovska, Scott Reddiex, Xiaofang Wang; Laboratory Scientists: Jeremy Anderson, Kristy Azzopardi, Vicki Bennett- Wood, Anna Czajko, Nadia Mazarakis, Conor McCafferty, Frances Oppedisano, Belinda Ortika, Casey Pell, Leena Spry, Ryan Toh, Sunitha Velagapudi, Amanda Vlahos, Ashleigh Wee-Hee; Biobanking: **Pedro Ramos**, Karina De La Cruz, Dinusha Gamage, Anushka Karunanayake, Isabella Mezzetti, Dr Benjamin Ong, Ronita Singh, Enoshini Sooriyarachchi;

Serology testing (VIDRL): **Dr Suellen Nicholson**, Natalie Cain, Rianne Brizuela, Han Huang;

Study Visit and Phone Call Team: **Veronica Abruzzo**, Morgan Bealing, Patricia Bimboese, Kirsty Bowes, Emma Burrell, Dr Joyce Chan, Jac Cushnahan, Hannah Elborough, Olivia Elkington, Kieran Fahey, Monique Fernandez, Catherine Flynn, Sarah Fowler, Marie Gentile Andrit, Bojana Gladanac, Catherine Hammond, Norine Ma, Sam Macalister, Emmah Milojevic, Jesutofunmi Mojeed, Jill Nguyen, Liz O'Donnell, Nadia Olivier, Isabelle Ooi, Stephanie Reynolds, Lisa Shen, Barb Sherry, Judith Spotswood, Jamie Wedderburn, Angela Younes;

Pharmacy Team: **Donna Legge,** Jason Bell, Jo Cheah, Annie Cobbledick, Kee Lim;

Immunisation Team: **Sonja Elia**, Lynne Addlem, Anna Bourke, Clare Brophy, Nadine Henare, Narelle Jenkins, Francesca Machingaifa, Skye Miller, Kirsten Mitchell, Sigrid Pitkin, Kate Wall;

Safety and Quality Monitoring Team: **Dr Paola Villanueva**, A/Prof Nigel Crawford, Dr Laure Pittet, Dr Wendy Norton;

Epworth Healthcare: **Dr Niki Tan**, Thilakavathi Chengodu, Diane Dawson, Victoria Gordon;

Monash Health: **Tony Korman**, Jess O'Bryan, Veronica Abruzzo;

MCRI Start-up Support Team: Sophie Agius, Dr Samantha Bannister, Jess Bucholc, Alison Burns, Beatriz Camesella, Prof John Carlin, Marianna Ciaverella, Maxwell Curtis, Stephanie Firth, Dr Christina Guo, Matthew Hannan, Erin Hill, Sri Joshi, Katherine Lieschke, Megan Mathers, Sasha Odoi, Ashleigh Rak, Dr Chris Richards, Leah Steve, Carolyn Stewart, Dr Eva Sudbury, Helen Thomson, Emma Watts, Fiona Williams, Angela Young;

Legal: **Penny Glenn**, Andrew Kaynes, Amandine Philippart De Floy App Development: Sandy Buchanan, Thijs Sondag, Ivy Xie;

Media and Communications: **Harriet Edmund**, Bridie Byrne, Tom Keeble, Belle Ngien, Fran Noonan, Michelle Wearing-Smith;

Orygen Volunteers: Alison Clarke, Pemma Davies, Oliver Eastwood, Alric Ellinghaus, Rachid Ghieh, Zahra Hilton, Emma Jennings, Athina Kakkos, Iris Liang, Katie Nicol, Sally O'Callaghan, Helen Osman, Gowri Rajaram, Sophia Ratcliffe, Victoria Rayner, Ashleigh Salmon, Angela Scheppokat, Aimee Stevens, Rebekah Street, Nicholas Toogood.

**Australia (New South Wales)**

Westmead Children’s Hospital: **A/Prof Nicholas Wood**, Twinkle Bahaduri, Therese Baulman, Jennifer Byrne, Candace Carter, Mary Corbett, Aiken Dao, Maria Desylva, Dr Andrew Dunn, Evangeline Gardiner, Rosemary Joyce, Dr Rama Kandasamy, Prof Craig Munns, Lisa Pelayo, Dr Ketaki Sharma, Katrina Sterling, Caitlin Uren; Westmead Hospital: Clinton Colaco, A/Prof Mark Douglas, Kate Hamilton; Sydney Children's Hospital: Dr Adam Bartlett, Dr Brendan McMullan, Dr Pamela Palasanthiran, Dr Phoebe Williams; Prince of Wales Hospital: Dr Justin Beardsley, Nikki Bergant, Renier Lagunday, Dr Kristen Overton, Prof Jeffrey Post; St Vincent’s Hospital Sydney: Dr Yasmeen Al-Hindawi, Sarah Barney, A/Prof Anthony Byrne, Lee Mead, Marshall Plit.

**Australia (South Australia)**

SAHMRI: **Prof. David Lynn**, Saoirse Benson, Dr Stephen Blake, Rochelle Botten, Tee Yee Chern, Georgina Eden, Liddy Griffith, Jane James, Dr Miriam Lynn, Angela Markow, Domenic Sacca, Dr Natalie Stevens, Prof. Steve Wesselingh; Royal Adelaide Hospital: Catriona Doran, Dr Simone Barry, Dr Alice Sawka; Women’s and Children’s Hospital: Dr Sue Evans, Louise Goodchild, Christine Heath, Meredith Krieg, Prof. Helen Marshall, Mark McMillan, Mary Walker.

**Australia (Western Australia)**

Perth Children’s Hospital/Telethon Kids Institute: **Prof Peter Richmond**, Nelly Amenyogbe, Christina Anthony, Annabelle Arnold, Beth Arrowsmith, Rym Ben-Othman, Sharon Clark, Jemma Dunnill, Nat Eiffler, Krist Ewe, Carolyn Finucane, Lorraine Flynn, Camille Gibson, Lucy Hartnell, Elysia Hollams, Heidi Hutton, Lance Jarvis, Jane Jones, Jan Jones, Karen Jones, Jennifer Kent, Prof Tobias Kollmann, Debbie Lalich, Wenna Lee, Rachel Lim, Sonia McAlister, Fiona McDonald, Andrea Meehan, Asma Minhaj, Lisa Montgomery, Melissa O’Donnell, Jaslyn Ong, Joanne Ong, Kimberley Parkin, Glady Perez, Catherine Power, Shadie Rezazadeh, Holly Richmond, Sally Rogers, Nikki Schultz, Margaret Shave, Patrycja Skut, Lisa Stiglmayer, Alexandra Truelove, Dr Ushma Wadia, Rachael Wallace, Justin Waring; Fiona Stanley Hospital: Michelle England, Erin Latkovic, A/Prof Laurens Manning; Sir Charles Gardiner: Dr Susan Herrmann, Prof Michaela Lucas.

**Brazil (Manaus)**

Manaus: **Dr Marcus Lacerda**, Paulo Henrique Andrade, Fabiane Bianca Barbosa, Dayanne Barros, Larissa Brasil, Ana Greyce Capella, Ramon Castro, Erlane Costa, Dilcimar de Souza, Maianne Dias, José Dias, Klenilson Ferreira, Paula Figueiredo, Thamires Freitas, Ana Carolina Furtado, Larissa Gama, Vanessa Godinho, Cintia Gouy, Daniele Hinojosa, Dr Bruno Jardim, Dr Tyane Jardim, Joel Junior, Augustto Lima, Bernardo Maia, Adriana Marins, Kelry Mazurega, Tercilene Medeiros, Rosangela Melo, Marinete Moraes, Elizandra Nascimento, Juliana Neves, Maria Gabriela Oliveira, Thais Oliveira, Ingrid Oliveira, Arthur Otsuka, Rayssa Paes, Handerson Pereira, Gabrielle Pereira, Christiane Prado, Evelyn Queiroz, Laleyska Rodrigues, Bebeto Rodrigues, Dr Vanderson Sampaio, Anna Gabriela Santos, Daniel Santos, Tilza Santos, Evelyn Santos, Ariandra Sartim, Ana Beatriz Silva, Juliana Silva, Emanuelle Silva, Mariana Simão, Caroline Soares, Antonny Sousa, Alexandre Trindade, Dr Fernando Val, Adria Vasconcelos, Heline Vasconcelos.

**Brazil (Mato Grosso do Sul)**

Mato Grosso do Sul: **Prof Julio Croda**, Carolinne Abreu, Katya Martinez Almeida, Camila Bitencourt de Andrade, Jhenyfer Thalyta Campos Angelo, Ghislaine Gonçalvez de Araújo Arcanjo, Bianca Maria Silva Menezes Arruda, Wellyngthon Espindola Ayala, Adelita Agripina Refosco Barbosa, Felipe Zampieri Vieira Batista, Fabiani de Morais Batista, Miriam de Jesus Costa, Dr Mariana Garcia Croda, Lais Alves da Cruz, Roberta Carolina Pereira Diogo, Rodrigo Cezar Dutra Escobar, Iara Rodrigues Fernandes, Leticia Ramires Figueiredo, Leandro Galdino Cavalcanti Gonçalves, Sarita Lahdo, Joyce dos Santos Lencina, Guilherme Teodoro de Lima, Larissa Santos Matos, Bruna Tayara Leopoldina Meireles, Debora Quadros Moreira, Lilian Batista Silva Muranaka, Adriely de Oliveira, Karla Regina Warszawski de Oliveira, Matheus Vieira de Oliveira, Prof Roberto Dias de Oliveira, Andrea Antonia Souza de Almeida dos Reis Pereira, Marco Puga, Caroliny Veron Ramos, Thaynara Haynara Souza da Rosa, Karla Lopes dos Santos, Claudinalva Ribeiro dos Santos, Dyenyffer Stéffany Leopoldina dos Santos, Karina Marques Santos, Paulo César Pereira da Silva, Paulo Victor Rocha da Silva, Débora dos Santos Silva, Patricia Vieira da Silva, Bruno Freitas da Rosa Soares, Mariana Gazzoni Sperotto, Mariana Mayumi Tadokoro, Daniel Tsuha, Hugo Miguel Ramos Vieira.

**Brazil (Rio de Janeiro)**

Rio de Janeiro: **Prof Margareth Maria Pretti Dalcolmo**, Cíntia Maria Lopes Alves da Paixão, Gabriela Corrêa E Castro, Simone Silva Collopy, Renato da Costa Silva, Samyra Almeida da Silveira, Alda Maria Da-Cruz, Alessandra Maria da Silva Passos de Carvalho, Rita de Cássia Batista, Maria Luciana Silva De Freitas, Aline Gerhardt de Oliveira Ferreira, Ana Paula Conceição de Souza, Paola Cerbino Doblas, Ayla Alcoforado da Silva dos Santos, Vanessa Cristine de Moraes dos Santos, Glauce Dos Santos, Dayane Alves dos Santos Gomes, Anderson Lage Fortunato, Adriano Gomes-Silva, Monique Pinto Gonçalves, Paulo Leandro Garcia Meireles Junior, Estela Martins da Costa Carvalho, Fernando do Couto Motta, Ligia Maria Olivo de Mendonça, Girlene dos Santos Pandine, Rosa Maria Plácido Pereira, Ivan Ramos Maia, Jorge Luiz da Rocha, João Victor Paiva Romano, Erica Fernandes da Silva, Marilda Agudo Mendonça Teixeira de Siqueira, Ágatha Cristinne Prudêncio Soares.

**The Netherlands**

UMC Utrecht: **Prof Marc Bonten,** Sandra Franch Arroyo, A/Prof Cristina Prat Aymerich, Henny Ophorst-den Besten, Anna Boon, Karin M Brakke, Axel Janssen, Marijke A.H. Koopmans, Toos Lemmens, Titia Leurink, Engelien Septer-Bijleveld, Kimberly Stadhouders, Dr Darren Troeman, Marije van der Waal, Marjoleine van Opdorp, Nicolette van Sluis, Beatrijs Wolters; Amphia Hospital: Prof Jan Kluytmans, Jannie Romme, Dr Wouter van den Bijllaardt, Linda van Mook, Dr M.M.L (Miranda) van Rijen; Rijnstate Hospital: P. M. G. Filius, Jet Gisolf, Frances Greven, Danique Huijbens, Dr Robert Jan Hassing, R. C. Pon, Lieke Preijers, J. H. van Leusen, Harald Verheij; Noord West Ziekenhuis: Dr Wim Boersma, Evelien Brans, Paul Kloeg, Kitty Molenaar-Groot, Nhat Khanh Nguyen, Dr Nienke Paternotte, Anke Rol, Lida Stooper; Radboud UMC: Helga Dijkstra, Esther Eggenhuizen, Lucas Huijs, Dr Simone Moorlag, Prof Mihai Netea, Eva Pranger, Dr Esther Taks, Dr Jaap ten Oever, Rob ter Heine; St Antonius Hospital: Kitty Blauwendraat, Dr Bob Meek, Isil Erkaya, Houda Harbech, Dr Nienke Roescher, Rifka Peeters, Menno te Riele, Carmen Zhou.

**Spain**

Mutua Terrassa University Hospital: Dr Esther Calbo, Cristina Badia Marti, Emma Triviño Palomares, Tomás Perez Porcuna; University Hospital Germans Trias I Pujol: Anabel Barriocanal, Ana Maria Barriocanal, Irma Casas, Jose Dominguez, Maria Esteve, Alicia Lacoma, Irene Latorre, Gemma Molina, Barbara Molina, Dr Antoni Rosell, Sandra Vidal; Hospital Virgen Macarena: Lydia Barrera, Natalia Bustos, Ines Portillo Calderón, David Gutierrez Campos, Jose Manuel Carretero, Angel Dominguez Castellano, Renato Compagnone, Encarnacion Ramirez de Arellano, Almudena de la Serna, Maria Dolores del Toro Lopez, Marie-Alix Clement Espindola, Ana Belen Martin Gutierrez, Alvaro Pascual Hernandez, Virginia Palomo Jiménez, Elisa Moreno, Nicolas Navarrete, Teresa Rodriguez Paño, Prof Jesús Rodríguez-Baño, Enriqueta Tristán, Maria Jose Rios Villegas; University Hospital Cruces: Atsegiñe Canga Garces, Erika Castro Amo, Raquel Coya Guerrero, Dr.Josune Goikoetxea, Leticia Jorge, Cristina Perez; Marqués de Valdecilla University Hospital: Dr María Carmen Fariñas Álvarez, Manuel Gutierrez Cuadra, Dr Francisco Arnaiz de las Revillas Almajano, Pilar Bohedo Garcia, Dr Teresa Giménez Poderos, Claudia González Rico, Blanca Sanchez, Olga Valero, Noelia Vega.

**United Kingdom**

University of Exeter/Exeter Clinical Trials Unit: **Prof John Campbell,** Anna Barnes, Dr Helen Catterick, Tim Cranston, Phoebe Dawe, Emily Fletcher, Liam Fouracre, Dr Alison Gifford, Neil Gow, John Kirkwood, Dr Christopher Martin, Dr Amy McAndrew, Marcus Mitchell, Georgina Newman, Dr Abby O'Connell, Jakob Onysk, Lynne Quinn, Dr Shelley Rhodes, Samuel Stone, Dr Lorrie Symons, Harry Tripp, Prof Adilia Warris, Darcy Watkins, Bethany Whale; St Leonard’s Practice: Dr Alex Harding, Gemma Lockhart, Dr Kate Sidaway-Lee; Ide Lane Surgery: Dr John Campbell, Dr Sam Hilton, Sarah Manton, Dr Daniel Webber-Rookes, Rachel Winder; Travel Clinic: James Moore; Royal Devon and Exeter NHS Foundation Trust: Freya Bateman, Dr Michael Gibbons, Dr Bridget Knight, Julie Moss, Dr Sarah Statton, Josephine Studham; Teign Estuary Medical Group/Glendevon Medical Practice: Lydia Hall, Will Moyle, Dr Tamsin Venton.

Supplementary Methods

**Break Through COVID-19 Infections Analysis Plan v1.0**

| **Objective** | **Estimand** |
| --- | --- |
| To determine if BCG vaccination compared with placebo reduces the incidence of i) symptomatic and ii) severe COVID-19 after receipt of COVID-19 vaccines* in healthcare workers recruited in stage 2† who receive a COVID-19 vaccine* | **Population**: Healthcare workers recruited in stage 2† who receive a COVID-19 vaccines* (subset of the ITT population)  **Outcomes:**  i) symptomatic COVID-19 after receipt of COVID-19 vaccines*  ii) severe COVID-19 after receipt of COVID-19 vaccines*  **Interventions:** BCG vs Placebo  **Handling of Intercurrent events:**  - any non-COVID-19 vaccine (treatment policy strategy)  - not receiving a COVID-19 vaccines* (principle stratification strategy)  **Summary Measure:** Hazard ratio |

* COVID-19 vaccines is defined as i) 14 days after 1 dose of Ad26.COV2., ChAdOx1, mRNA-1273, BNT162b2, or CoronaVac and ii) 14 days after receiving 1 dose of Ad26.COV2. or 2 doses of ChAdOx1, mRNA-1273, BNT162b2, CoronaVac or 2 doses of a mix of the 4 latter (heterologous primary vaccination). † Stage 1 healthcare workers were recruited only in Victoria and Western Australia, both of which states had almost negligible COVID-19 exposure risk during the trial period (30th Mar 2020 to 13th May 2020). In light of this, the overwhelming majority of Stage 1 blood samples are likely to be seronegative. Moreover, with a low prevalence of COVID-19, there is a high probability that positive SARS-CoV-2 serology results are false positive. For these reasons, in December 2021 the BRACE team decided it was not justifiable to devote extra resources to the data cleaning of potential COVID-19 episodes and to testing SARS-CoV-2 serology for all participants in Stage 1. All COVID-19 related outcomes are reported in Stage 2 participants only.

*Defining the Outcome*

Symptomatic and severe COVID-19 will be defined as per the primary outcomes for the trial (see Statistical Analysis Plan v2.0- Section 4.2) although for this outcome the period over which the event can occur is different to that in the primary outcome. For this outcome, a person is only at risk for this event 14 days after receiving

1. 1 dose of Ad26.COV2., ChAdOx1, mRNA-1273, BNT162b2, or CoronaVac
2. 1 dose of Ad26.COV2. or 2 doses of ChAdOx1, mRNA-1273, BNT162b2, CoronaVac or 2 doses of a mix of the 4 latter (heterologous primary vaccination).

NB: Definition i correspond to the first dose of any COVID-19-specific vaccine, and definition ii correspond to completing primary vaccination series. Heterologous primary vaccination series are also considered as complete (e.g. if 1st dose of mRNA-1273 is followed by a dose of BNT162b2). As the Ad26.COV2 schedule is only a single dose, vaccination is considered to be complete after the receipt of the first dose.

*Assumptions*

In conducting this analysis, we assume that the receipt of COVID-19 vaccine is unrelated to receipt of the study intervention (BCG/Placebo). Preliminary investigations of time to receipt of COVID-19 vaccine by treatment arm did not show any difference in time to first dose, suggesting that this assumption may be appropriate. We will examine time to receipt of COVID-19 vaccines (define both as i/ii, above) as well as type of vaccine received by arm prior to undertaking this analysis to confirm that no obvious differences by treatment arm (BCG/Placebo) are evident. If there are any clear differences by treatment arm, we will not compare treatment arms but report the outcome descriptively only.

By definition, participants who do not receive COVID-19 vaccines (definition i/ii, above) during the follow-up period will not become at risk for an event. The non-receipt of a COVID-19 vaccines will be handled via a principle stratification strategy, e.g. examining the effect of BCG/ placebo in a stratum of participants who would not receive a first dose or primary series of COVID-19 vaccines. Very few participants would be expected to meet these conditions, as most healthcare workers at BRACE sites received a primary series of COVID-19 vaccines during the trial. Under the previously stated assumption that COVID-19 vaccine receipt is unrelated to treatment arm, we assume that excluding participants without a first dose or primary series of COVID vaccines will be valid for the handling of this intercurrent event under a principle stratification strategy. Selection bias of participants into the analysis could however be an issue and will need to be considered when interpreting findings.

There could be differences by treatment arm in COVID-19 infection prior to receiving COVID-19 vaccines which could influence this analysis. By 6 months, 135 participants in the BCG group had COVID-19 infection compared to 107 in the placebo group prior to receipt of COVID-19 vaccine (HR 1.23 (95%CI 0.96 to 1.59)). We will adjust for COVID-19 infection prior to COVID-19 vaccine receipt in our analyses.

*Analysis*

As per the primary outcome, the proportion of participants meeting the outcome will be determined using a time to event analysis. In this analysis, time will be calculated from date of receiving a COVID-19 vaccines (i/ii definition, as above) and participants will be censored at the earlier of:

**[A]** day 365 of their participation in the trial or

**[B]** their last entered date prior to which there are more than 3 consecutive days of missing data which aren’t ruled out by negative serology (as a negative serology can reasonably exclude that a COVID-19 episode happened during the period of missing data).

**[C]** their first day with symptoms for their first episode of illness with trigger/severe symptoms, which the algorithm in Figure 4 cannot ascertain be a COVID-19 episode (categorised as missing in Statistical Analysis Plan v2.0- Fig.4)

unless the definition of the outcome is met and first day with symptoms for their outcome precedes all the events above, [A] – [C].

Symptomatic COVID-19 and severe COVID-19 after receipt of COVID-19 specific vaccine (i/ii definition, as above) will be compared by treatment arm in healthcare workers recruited in Stage 2 using a hazard ratio. This will be estimated using a cox regression analysis model adjusted for stratification factors used during randomisation (age group, presence of comorbidity, geographical location -Europe/Australia/South America), type of vaccine received (i: Ad26.COV2./ChAdOx1, mRNA-1273/BNT162b2/CoronaVac, ii: Ad26.COV2/ChAdOx1 + ChAdOx1/mRNA-1273 + mRNA-1273/BNT162b2 + BNT162b2/CoronaVac + CoronaVac/heterologous primary vaccination (mixed two doses)) and COVID-19 infection prior to COVID-19 vaccine receipt (yes/no). For participants who were randomised in the incorrect stratum, the correct stratum will be used as covariate in the model. We will also descriptively report the number of events by type of vaccine received and treatment arm.

A Kaplan-Meier survival curve will also be presented by treatment arm.

The proportional hazards assumption will be checked when running these analyses.

Supplementary Table 1. Additional Baseline characteristics and COVID-19 vaccinations

|  | **Intention-to-treat**  **population** | |  | **Modified intention-to-treat population** | |
| --- | --- | --- | --- | --- | --- |
|  | Placebo | BCG |  | Placebo | BCG |
| **Baseline characteristic** | N = 1989 | N = 1999 |  | N = 1683 | N = 1703 |
| Workplace |  |  |  |  |  |
| Emergency Department | 120 (6%) | 100 (5%) |  | 89 (5%) | 77 (5%) |
| Intensive Care Unit / High Dependency Unit | 112 (6%) | 127 (6%) |  | 98 (6%) | 112 (7%) |
| Operating Theatre | 83 (4%) | 70 (4%) |  | 75 (4%) | 68 (4%) |
| General ward | 216 (11%) | 235 (12%) |  | 182 (11%) | 210 (12%) |
| Pharmacy | 77 (4%) | 73 (4%) |  | 52 (3%) | 46 (3%) |
| Other ward/area | 1012 (51%) | 1014 (51%) |  | 850 (51%) | 854 (50%) |
| Paramedic / Ambulance | 24 (1%) | 24 (1%) |  | 23 (1%) | 23 (1%) |
| Aged care facility | 33 (2%) | 31 (2%) |  | 29 (2%) | 29 (2%) |
| Practice outside of hospital | 312 (16%) | 325 (16%) |  | 285 (17%) | 284 (17%) |
| Work role |  |  |  |  |  |
| Nurse/Midwife | 370 (19%) | 398 (20%) |  | 326 (19%) | 359 (21%) |
| Doctor | 198 (10%) | 208 (10%) |  | 188 (11%) | 197 (12%) |
| Allied Health | 432 (22%) | 422 (21%) |  | 374 (22%) | 354 (21%) |
| PSA/hospital maintenance | 318 (16%) | 328 (16%) |  | 233 (14%) | 248 (15%) |
| Administrative/clerical staff | 308 (15%) | 309 (15%) |  | 257 (15%) | 258 (15%) |
| Other | 81 (4%) | 83 (4%) |  | 68 (4%) | 69 (4%) |
| Paramedic | 30 (2%) | 29 (1%) |  | 28 (2%) | 29 (2%) |
| Carer | 21 (1%) | 21 (1%) |  | 19 (1%) | 16 (1%) |
| Dentist/dental therapy | 42 (2%) | 29 (1%) |  | 33 (2%) | 23 (1%) |
| Community Health Agent | 101 (5%) | 86 (4%) |  | 87 (5%) | 77 (5%) |
| Scientist (medical/research) | 88 (4%) | 86 (4%) |  | 70 (4%) | 73 (4%) |
| Confirmed COVID-19 patients within department |  |  |  |  |  |
| No | 724 (36%) | 724 (36%) |  | 639 (38%) | 631 (37%) |
| Yes | 1265 (64%) | 1275 (64%) |  | 1044 (62%) | 1072 (63%) |
| Smoking |  |  |  |  |  |
| No | 1778 (89%) | 1803 (90%) |  | 1499 (89%) | 1527 (90%) |
| Yes, rarely (1 or 2 cigarettes a month) | 46 (2%) | 37 (2%) |  | 39 (2%) | 29 (2%) |
| Yes, occasionally (1 or 2 cigarettes a week) | 38 (2%) | 42 (2%) |  | 30 (2%) | 39 (2%) |
| Yes, regularly | 127 (6%) | 117 (6%) |  | 115 (7%) | 108 (6%) |
| Prior latent TB diagnosis |  |  |  |  |  |
| No | 1972 (99%) | 1981 (99%) |  | 1666 (99%) | 1686 (99%) |
| Yes | 8 (0%) | 11 (0%) |  | 8 (0%) | 11 (1%) |
| Not sure | 9 (0%) | 7 (0%) |  | 9 (0%) | 6 (0%) |
| Prior positive tuberculin skin test or Mantoux test |  |  |  |  |  |
| No | 1760 (88%) | 1793 (90%) |  | 1464 (87%) | 1508 (89%) |
| Yes | 122 (6%) | 101 (5%) |  | 114 (7%) | 94 (6%) |
| Not sure | 107 (5%) | 105 (5%) |  | 105 (6%) | 101 (6%) |
| Presence of comorbidities - as per randomisation strata (excludes obesity) | |  |  |  |  |
| No comorbidities | 1600 (80%) | 1597 (80%) |  | 1350 (80%) | 1345 (79%) |
| Comorbidities | 389 (20%) | 402 (20%) |  | 333 (20%) | 358 (21%) |
| Number of comorbidities (including BMI≥30kg/m^2^) |  |  |  |  |  |
| None | 1600 (80%) | 1597 (80%) |  | 1350 (80%) | 1345 (79%) |
| 1 | 349 (18%) | 354 (18%) |  | 296 (18%) | 319 (19%) |
| 2 | 37 (2%) | 47 (2%) |  | 34 (2%) | 38 (2%) |
| 3 | 3 (0%) | 1 (0%) |  | 3 (0%) | 1 (0%) |
| **COVID-19 vaccination** |  |  |  |  |  |
| Type of primary series of vaccination(s) received (first dose/second dose) | |  |  |  |  |
| ChAdOx1/ChAdOx1 | 579 (32%) | 617 (33%) |  | 498 (32%) | 526 (33%) |
| BNT162b2/BNT162b2 | 397 (22%) | 412 (22%) |  | 364 (23%) | 380 (24%) |
| mRNA-1273/mRNA-1273 | 110 (6%) | 100 (5%) |  | 103 (7%) | 99 (6%) |
| CoronaVac/CoronaVac | 670 (36%) | 643 (35%) |  | 521 (33%) | 497 (31%) |
| Ad26.COV2.S | 71 (4%) | 73 (4%) |  | 66 (4%) | 70 (4%) |
| Heterologous | 11 (0%) | 9 (0%) |  | 10 (1%) | 7 (0%) |
| missing | 151 | 145 |  | 121 | 124 |

Supplementary Table 2. COVID-19 by 12 months following randomisation, inclusive of adjustment for sex

|  | **Placebo** | **BCG** | **Difference^a^** | **P-value** |
| --- | --- | --- | --- | --- |
|  | N=1683 | N=1703 | (BCG-Placebo) |  |
| **Symptomatic COVID-19 by 12 months** | 308 | 356 |  |  |
| Adjusted estimated percent, with 95% CI^a^ | 19.5% (17.6 to 21.5) | 22.6% (20.6 to 24.6) | 3.1% (0.4 to 5.8) | 0.03 |
| **Severe COVID-19 by 12 months** | 158 | 182 |  |  |
| Adjusted estimated percent, with 95% CI^a^ | 9.6% (8.2 to 11.0) | 11.0% (9.5 to 12.5) | 1.4% (-0.7 to 3.5) | 0.18 |

^a^Adjustment for a priori baseline covariates included sex in addition to stratification factors.

Supplementary Table 3. Diabetes subgroup analysis

| **Outcome** | **Placebo** | **BCG** | **Difference^a^** | **P-value for** |
| --- | --- | --- | --- | --- |
|  | N=1683 | N=1703 | (BCG-Placebo) | **interaction** |
| **Symptomatic COVID-19 by 12 months** |  |  |  | 0.06 |
| *No diabetes,* n/total n | 155/1616 | 168/1651 |  |  |
| Adjusted estimated percent, with 95% CI^a^ | 19.7% (17.8 to 21.6) | 22.1% (20.1 to 24.1) | 2.4% (-0.3 to 5.1) |  |
| *Diabetes****,*** n/total n^g^ | 10/67 | 19/52 |  |  |
| *Type I* | 2 (20%) | 4 (21%) |  |  |
| *Type II* | 8 (80%) | 15 (79%) |  |  |
| Adjusted estimated percent, with 95% CI^a^ | 15.9% (6.9 to 25.0) | 38.7% (25.4 to 52.0) | 22.7% (6.8 to 38.7) |  |
| **Severe COVID-19 by 12 months** |  |  |  | 0.003 |
| *No diabetes,* n/total n | 298/1616 | 337/1651 |  |  |
| Hospitalised | 7 (5%) | 9 (5%) |  |  |
| Non-ambulant | 34 (22%) | 25 (15%) |  |  |
| Too unwell to work | 114 (74%) | 134 (80%) |  |  |
| Adjusted estimated percent, with 95% CI^a^ | 9.9% (8.5 to 11.4) | 10.4% (9.1 to 12.0) | 0.6% (-1.5 to 2.7) |  |
| *Diabetes****,*** n/total n^g^ | 3/67 | 14/52 |  |  |
| *Type I* | 1 (33%) | 4 (29%) |  |  |
| *Type II* | 2 (67%) | 10 (71%) |  |  |
| Hospitalised | 0 (0%) | 2 (14%) |  |  |
| Non-ambulant | 0 (0%) | 1 (7%) |  |  |
| Too unwell to work | 3 (100%) | 11 (79%) |  |  |
| Adjusted estimated percent, with 95% CI^a^ | 4.3% (1.4 to 13.0) | 29.1% (19.3 to 43.8) | 24.8% (11.9 to 37.8) |  |

^a^ Adjusted for stratification factors

# Supplementary Figure 1

**Supplementary Figure 1.** Breakthrough COVID-19 by 12 months following randomisation, by treatment arm.

Kaplan–Meier curves of time from COVID-19 vaccination with 95% confidence intervals in shaded areas (unadjusted analyses in the intention‐to‐treat population ITT). Panels A and B present COVID‐19 after first dose of a COVID-19 vaccination and panels B and D show COVID‐19 after the primary course (2 doses, except for recipients of Ad26.COV2.S who only require one dose) of COVID-19 vaccination. Panels A and C present symptomatic COVID‐19 and panels B and D present severe COVID‐19.
